# Quantitative Mass Spectrometry Imaging of Bleomycin in Skin Using a Mimetic Tissue Model for Calibration

**DOI:** 10.3390/ph15121583

**Published:** 2022-12-19

**Authors:** Andreas Traberg, Fernanda E. Pinto, Anders C. N. Hansen, Merete Haedersdal, Catharina M. Lerche, Christian Janfelt

**Affiliations:** 1 Department of Pharmacy, Faculty of Health and Medical Sciences, University of Copenhagen, Universitetsparken 2, 2100 Copenhagen, Denmark; 2 Department of Dermatology, Copenhagen University Hospital, Bispebjerg, 2400 Copenhagen, Denmark

**Keywords:** MALDI-MS, drug distribution, quantitation, mass spectrometry imaging

## Abstract

The aim of Quantitative mass spectrometry imaging (Q-MSI) is to provide distribution analysis and quantitation from one single mass-spectrometry-based experiment, and several quantitation methods have been devised for Q-MSI. Mimetic tissue models based on spiked tissue homogenates are considered one of the most accurate ways to perform Q-MSI, since the analyte is present in a well-defined concentration in a sample matrix highly similar to the one of the unknown sample to be analyzed. The delivery of drugs in skin is among the most frequent types of pharmaceutical MSI studies. Here, a mimetic tissue model is extended for use on the skin, which, due to its high collagen content, is different from most other tissue as the homogenates become extremely viscous. A protocol is presented which overcomes this by the addition of water and the handling of the homogenate at an elevated temperature where the viscosity is lower. Using a mimetic tissue model, a method was developed for the quantitative imaging of bleomycin in skin. To compensate for the signal drift and the inhomogeneities in the skin, an internal standard was included in the method. The method was tested on skin from a pig which had had an electropneumatic injection of bleomycin into the skin. Quantification was made at several regions in a cross section of the skin at the injection site, and the results were compared to the results of a quantitative LC-MS on a neighboring tissue biopsy from the same animal experiment. The overall tissue concentration determined by the LC-MS was within the range of the different regions quantified by the Q-MSI. As the model provides the results of the same order of magnitude as a LC-MS, it can either be used to replace LC-MS in skin studies where MSI and LC-MS are today carried out in combination, or it can add quantitative information to skin studies which are otherwise carried out by MSI alone.

## 1. Introduction

Mass spectrometry imaging (MSI) is a family of analytical techniques that rely on mass spectrometry data in the generation of compound-specific images. MSI exists in different versions where Matrix assisted laser desorption ionization (MALDI) [1] and Desorption electrospray ionization (DESI) [2,3] are the two most commonly used ionization techniques in the MSI analysis of biological samples.

MSI is used in a variety of fields such as drug development [4], cancer research [5], microbiology [6] and plant biology [7]. In most studies, MSI has been applied in a mostly qualitative fashion—through the generation of a number of compound-specific images, MSI provides information about the distribution of exogenous compounds (e.g., drugs) as well as endogenous compounds (lipids, peptides, protein, carbohydrates and various metabolites) of which some may be used as tissue markers. Typically, the results do not provide information about the relative abundances of the detected compounds, nor do they provide any absolute tissue concentrations. The challenges here are that different compounds have different ionization efficiencies, and that ionization in an MSI experiment takes place in situ among and in competition with all the other compounds present in the sample (separation such as in conventional bioanalysis by LC-MS is not a possibility), thus causing the degree of *ion suppression* to vary across the sample surface. The first challenge is handled as in most other forms of quantitative MS by making an individual calibration curve for every compound to be detected, while the second challenge is handled by either assessing to what degree the ion suppression varies across the surface or by introducing an internal standard whose signal can be applied to even out such variations.

A number of ways to prepare the calibration curves in quantitative MSI (Q-MSI) have been devised, and the two most common calibration methods in MSI are based on the deposition of the standards on the control tissue and by the preparation of spiked tissue homogenates, respectively [8,9]. The deposition of the standards, typically 1–2 µL droplets on the control tissue, has the advantage that it is relatively quickly performed, which is an advantage in particular when the scope of the quantitative efforts is limited, e.g., the determination of the limits of the detection. It does, however, rely on two necessary assumptions: that the deposited compound distributes homogenously through the entire thickness of the tissue below the droplet (an average tissue concentration is calculated based on how large an area is covered by the deposited droplet), and that a compound deposited on top of the tissue undergoes the same interactions and ion suppressions from the various molecules in the tissue as if the compound were a more integral part of the tissue. The first assumption has been shown to be fair in the cases of relatively thin cryo-sections [10] while the second assumption may not be on quite as solid ground as the first one.

The spiked tissue homogenates, also known as a *mimetic tissue model*, were introduced as an MSI calibration model by Castellino and co-workers in 2013 [9]. The model is based on the homogenization of the control tissue, which is spiked to well-defined tissue concentrations and frozen in blocks, which are subsequently cryo-sectioned in the same thickness as the unknown samples. Compared with the droplet deposition model, the spiked tissue homogenates take a significantly longer time to prepare, but once a block of tissue homogenates has been prepared, a large number of identical sections can be made. This is an advantage in large studies involving several samples to by studied by Q-MSI. Furthermore, it is a great asset in comparison with different MSI instrumentation or analysis methods that a large number of identical and very well-defined, yet realistic samples with the drug in the presence of a sample matrix can be produced. Even more importantly, the model is considered to reproduce the interactions that the analyte of interest undergoes in the unknown samples very well, hence its name being a mimetic tissue model.

The mimetic tissue model was originally developed using liver tissue [9], and since then it has also been used on kidney [8,11] and brain tissue [8]. In one of these studies, it was also briefly used on skin, but only with the tissue homogenates of rat liver tissue serving as “surrogates” [8]. To our knowledge, however, it has not yet been made in skin tissue, although dermatology and cutaneous drug delivery is a major field of the application of MSI [12]. Moreover, skin is so different from most other tissues in its composition that it would be relevant to see if a real mimetic tissue model could be made for skin instead of a surrogate model. Over the years, several quantitative MSI studies have been published on various types of skin, such as human skin [13], porcine skin [14] and skin equivalent models [15], but in all the studies the quantification was based on different variants of the droplet deposition model, including use of the so-called *tissue extinction coefficient* [16]. In the present study, we present an approach to the Q-MSI of drugs in porcine skin based on a mimetic tissue model. Compared with most other tissues (liver, brain, kidney, etc.), skin is very different, due in particular to its high content of collagen, and a number of modifications were necessary in the preparation of the spiked tissue homogenates. The method was tested in a drug distribution study from an in vivo experiment on the delivery of the anti-cancer drug bleomycin using a novel injection technique known as an electropneumatic injection (EPI) and was compared to the results obtained by LC-MS on similar biopsies.

## 2. Results

### 2.1. Development of a Mimetic Tissue Model for Skin

The starting point of the design of a mimetic tissue model was the approach by Barry et al. [8,17], which was modified to accommodate the special properties of skin tissue. What makes skin different from most other tissues is its very high contents of collagen, which is a protein (known from gelatine) that produces hydrogels when in contact with water [18]. This is indeed what happens during the homogenization of the skin, when the skin turns into a viscoelastic solid upon a few minutes of bead beater homogenization. However, the viscosity of the hydrogel decreases at elevated temperatures, and we found it less difficult to work with the skin homogenate at temperatures in the range of 40–52 °C, as measured on the homogenizer thermometer. Another difference between the skin and most other tissues is its low content of water [19] compared to all other organs [20]; this also contributes to much more viscous tissue homogenates than what are typically seen in studies with a mimetic tissue model. Thus, as a consequence of the viscoelastic solid, which the homogenate turned into, we found it necessary to add as much as 150% water in order to be able to produce a homogenate. In addition, to deposit the still quite viscous skin homogenate at the bottom of the plastic syringe (used as a mold for the calibrants), we used a hand centrifuge to briefly spin the syringe upon the addition of the homogenate. We found the hand centrifuge easier to control than a conventional motorized centrifuge, which can cause phase separation. Another deviation from the approach of Barry et al. was that instead of using one relatively large syringe for the entire calibration row (seven homogenates), we used four smaller syringes containing two homogenates each. This meant that four homogenates could be made in parallel, reducing the freezing steps when the next homogenate was added. Furthermore, to ease the definition of the regions of interest (ROI) of the different concentrations in the subsequent data analyses, we added a thin layer of water (equivalent to 50 µL) on top of the first homogenate. This layer of water was allowed to freeze before the second homogenate was added to the syringe. Finally, the four homogenate cylinders were assembled in a silicone mold and a 5% gel of carboxymethyl cellulose was poured over the cylinder, so that a block containing eight separate spiked skin homogenates and a blank one could be frozen for cryo-sectioning. Compared to using one syringe for all eight homogenates, this would provide cryo-sections of eight calibrants, which were shorter but higher (in image aspect ratio).

**Figure 1:**
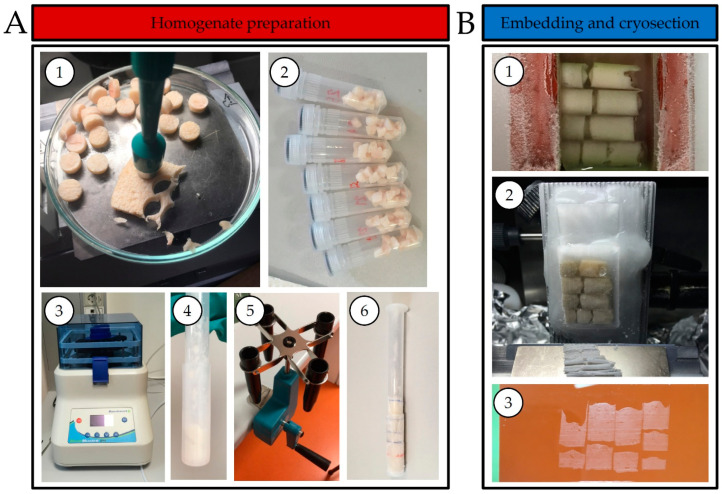
Mimetic tissue model preparation. (**A**) Homogenate preparation: punching of 8 mm biopsies (1), cutting in smaller pieces for a homogenization tube with water and 2 steel homogenization beads (2), homogenization (3), deposition of homogenate into a dosing pipette (4), centrifugation (5) and the frozen dosing pipette with homogenate (6). (**B**) Embedding and cryo-sectioning of the homogenate: embedding of the homogenates in a 5% CMC gel (1), cryo-sectioning of mimetic tissue model (2) and final section of the mimetic tissue model (3).

The entire process is shown in Figure 1, and additional details about the preparation of the calibrants, including the spiking with the standards, are provided in the Materials and Methods section.

### 2.2. Introduction of the Internal Standard

**Figure 2:**
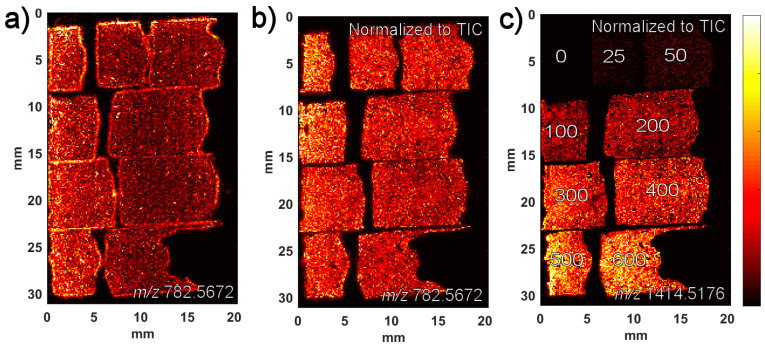
MALDI images of the mimetic tissue model. (**a**) PC(34:1) (*m/z* 782.5672) with no normalization. (**b**) PC(34:1) (*m/z* 782.5672) with normalization to TIC. (**c**) Bleomycin A2 (*m/z* 1414.5176) with normalization to TIC.

**Figure 3:**
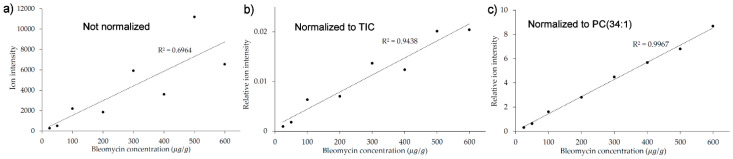
Standard curves generated using different types of normalization. (**a**) Non-normalized. (**b**) Normalized to TIC. (**c**) Normalized to reference peak of the endogenous lipid (PC(34:1)).

A series of homogenates spanning a bleomycin concentration range of 25–600 µg/g were prepared, and the block was imaged by MALDI-MSI; the resulting images are seen in Figure 2. As seen in the image in Figure 2a of the endogenous lipid PC(34:1) (*m/z* 782.5672), there is a downhill drift in the ion signal at the right side of the image despite a homogeneous distribution of the lipid. The drift is probably due to a change in the laser focus over the 2 cm width of the image, a drift which is likely to be present also in the signal of bleomycin (Figure 2c). Such variations can often be taken into account by normalizing the ion signal to the total ion current (TIC) rather than using the raw ion signal. As seen in Figure 2b, the TIC normalization does improve the homogeneity of the image to some extent, but there is still some bias in the image. This undesired effect and its consequences become clearer when the average ion intensities are calculated for each concentration region and the calibration curves are plotted with and without normalization to the TIC, respectively, as seen in Figure 3. The linearity of the calibration curve is better with the TIC normalization (Figure 3b) than without (Figure 3a), but still not very good. However, an alternative to normalizing to the TIC would be to normalize to the signal of an endogenous lipid, since the lipid is homogenously present throughout the tissue, and its signal will undergo the same variations as the bleomycin signal. As seen in Figure 3c, such normalization to the *m/z* 782.5672 peak of PC(34:1) makes a significant improvement to the linearity of the calibration curve, thus demonstrating the potential of an internal standard. Unfortunately, PC(34:1) is only homogeneously present in tissue homogenates and not in the real skin samples in which we intend to quantify bleomycin. Therefore, another compound which is homogenously distributed must be used. A solution could be to deposit an internal standard on the calibrants as well as on the unknown samples.

**Figure 4:**
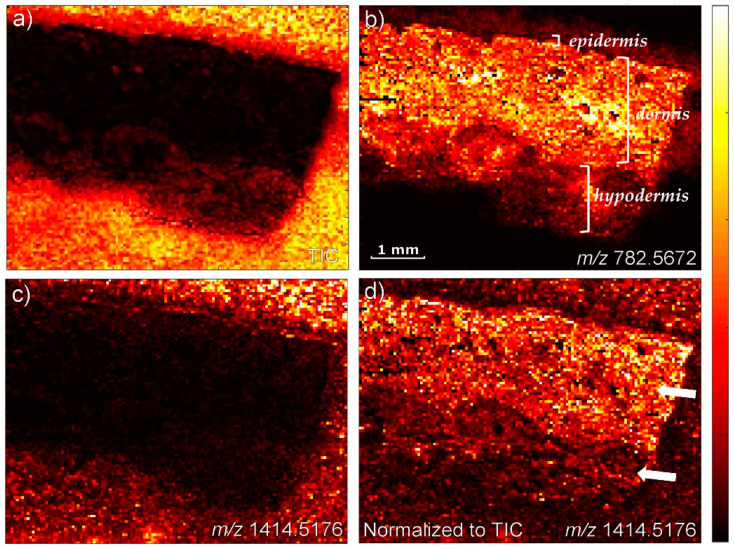
MALDI images of porcine skin after homogenous deposition of a layer of bleomycin. (**a**) TIC image. Outside the tissue, MALDI matrix peaks contribute greatly to the TIC. (**b**) The endogenous lipid PC(34:1) (*m/z* 782.5672), normalized to the TIC. The different histological regions in the skin are marked. (**c**) Bleomycin A2, not normalized (*m/z* 1414.5176). (**d**) Bleomycin A2, normalized to TIC (*m/z* 1414.5176). The image was acquired with 50 µm pixel size.

There are other reasons to consider the use of an internal standard, such as the heterogeneity of skin. The skin homogenate, which constitutes the matrix in our calibration model, is much closer to real skin sections than the various surrogate homogenates, but it still only represents an average of the different regions of the skin (stratum corneum, epidermis, dermis, hypodermis). The bleomycin may thus undergo different degrees of ion suppression in the different regions in the skin, an effect which the internal standard will be able to correct for. Such potential differences in ion suppression were tested for in an experiment where the control skin was coated with a layer of bleomycin and subsequently imaged. The results of this experiment are seen in Figure 4. Figure 4c shows a generally lower signal of bleomycin in the tissue than outside the tissue, which is well in line with the tissue causing the ion suppression of the signal from the bleomycin. In the absence of an ion suppression, the bleomycin signal should be homogenous throughout the image. As seen in Figure 4d, normalization to the TIC does not fully account for these differences, as the dermis appears with a higher bleomycin signal than the hypodermis, despite the amounts being similar due to the homogenous coating. Based on this, it was decided to introduce an internal standard which would undergo the same variations in ion suppression, laser focus and other phenomena affecting the signal. Since bleomycin is a natural product (produced in the *Streptomyces verticillus* bacteria), highly similar structure analogues are not readily found, and reserpine was chosen as an internal standard given its availability and relatively high molecular weight compared to most other small molecules.

### 2.3. Application of the Developed Mimetic Tissue Model in a Quantitative Study

**Figure 5:**
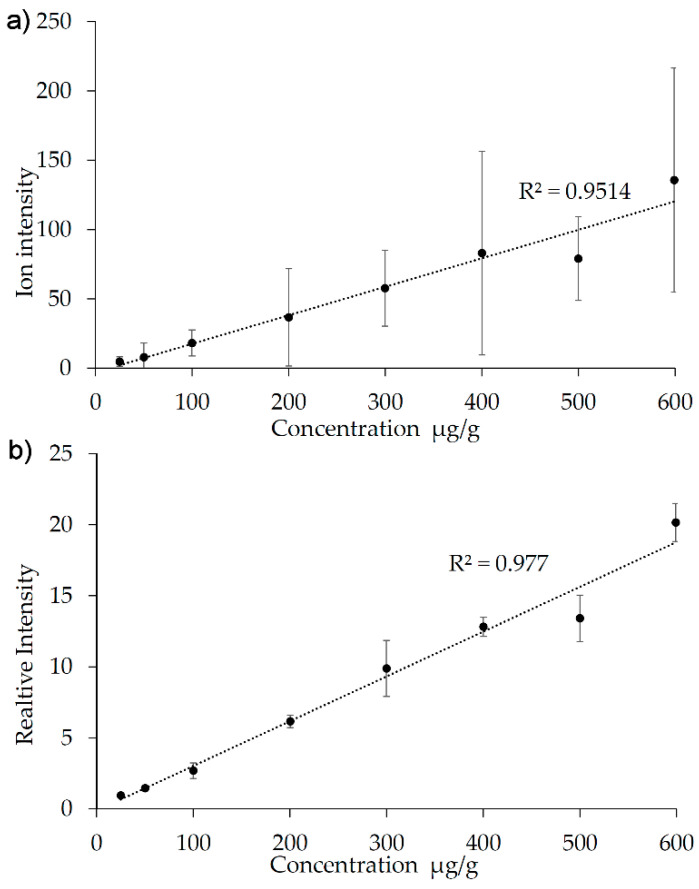
Calibration curve based on a mimetic tissue model in porcine skin with and without normalization to the internal standard. (**a**) Without normalization. (**b**) With normalization to the reserpine signal at *m/z* 609.2804. The error bars show the standard variation calculated from measurements on neighboring sections of the calibration block on three consecutive days.

The cryo-section of the mimetic tissue model had a size of 28 × 17 mm. Acquiring an image in high resolution would take an unreasonably long time to acquire, considering that only intensities, but no spatial details, would be needed from such an image. The sample, on the other hand, was a cross section of porcine skin, and here a spatial resolution of 50 µm was appropriate. Since a 50 µm pixel size is easily obtained on an AP-SMALDI5 system without any risk of oversampling, the intensities would not be affected by choosing a greater pixel size as long as no changes were made to the laser energy. It was therefore decided to image the sample at a 50 µm pixel size (resulting in an acquisition time of 127 min) and the mimetic tissue model at a 150 µm pixel size (resulting in an acquisition time of 272 min). The two images were run separately, but immediately after one another, and both samples underwent the same preparation with the internal standard and the matrix deposition. Figure 5 shows the resulting calibration curve with and without the use of reserpine as internal standard. The calibration curve was imaged and generated on three consecutive days from three neighboring cryo-sections (with fresh sample preparation each day), and the relative standard deviation of the slope of the three calibration curves was 7%. For each concentration step, the standard deviation was calculated and specified with error bars in the two calibration curves. As observed, the use of the internal standard provides a significant reduction of the day-to-day variation. When the internal standard is used, the relative standard deviation is in the range of 5–25% for the individual concentration steps. The lowest step in the calibration curve (*c* = 25 µg/g) was clearly above the LOD, which based on the signal intensities is estimated to be in the range of 5–15 µg/g.

**Figure 6:**
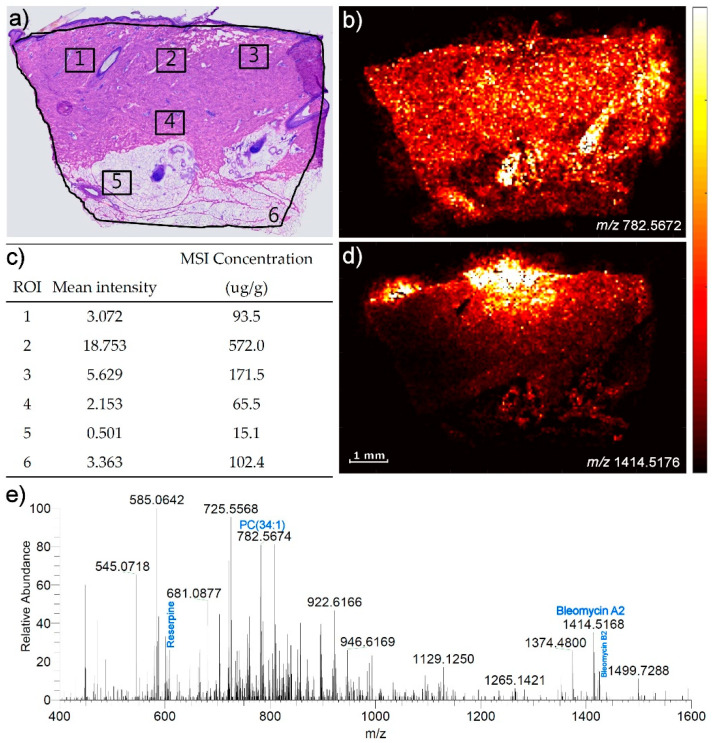
Quantitative MALDI imaging of a cross section of a skin biopsy from a pig, which was given an electropneumatic injection of bleomycin. (**a**) Microscope image of a neighboring H&E stained cryo-section. Squares mark the regions, which were later quantified. (**b**) MALDI-MS image of the endogenous lipid PC(34:1) (*m*/*z* 782.5682). (**c**) Table with results of quantification of bleomycin in the regions of interest indicated in a). (**d**) Bleomycin A2 (*m/z* 1414.51766), normalized to the signal of the internal standard reserpine. (**e**) Example of a mass spectrum extracted in ROI#4.

Figure 6 shows the application of the calibration curve on a cross section of a skin biopsy from a pig that has been given an electropneumatic injection (EPI) of bleomycin where the quantification is made in several different regions and at several different depths in the skin. This biopsy was part of a previous study where a quantitative LC-MS was performed on a neighboring biopsy from skin undergoing the same bleomycin exposure, and in that study the average tissue concentration of bleomycin in the whole biopsy was measured to be 63.5 µg/g [21]. The figure also shows an example of a mass spectrum from a region in the image. As seen in the table in Figure 6c, the concentrations were determined in several regions (1–6) in the skin resulting in concentrations in the range of 15.1–572.0 µg/g. The experiment was carried out in duplo to assess the reproducibility of the quantification. The cryo-section used for the second experiment was from the same biopsy, but not a neighboring section, so in principle the sections could be slightly different in terms of their bleomycin concentrations. The new regions (1–6) were marked in the second section to resemble regions 1–6 in Figure 6a of the first section as closely as possible. For regions 1–6, deviations between the two quantifications experiments of 2%, 24%, 19%, 28%, 12% and 10%, respectively, were found. Among these regions, region 6 represents the average of the entire section and was found to deviate by 10% between the two measured sections.

## 3. Discussion

### 3.1. Development of the Mimetic Tissue Model

This is, to our knowledge, the first time that an amount of water corresponding to 150% has been added in the preparation of a mimetic tissue model, and the possible implications of this addition of water are worth considering. While Barry et al. did not add any liquid (other than the standard solution used to spike the homogenates) when they made homogenates of liver, brain and kidney tissue [8], we had previously added 20% water to produce homogenates of heart, liver, lung, kidney and brain tissue, but in the same study, we also showed that the addition of water does not have any consequences for the quantification as long as it is accounted for in the calculation of the tissue drug concentration [10]. Prior to imaging, the sample is thawed in a vacuum desiccator, a step in which all the added water evaporates along with the natural content of water in the skin, leaving the mimetic tissue model in the same state as a skin section with regard to their contents of water. As noted in our previous study, the addition of water implies that when the mimetic tissue model is sectioned in the same thickness as the unknown samples, there will, upon the evaporation of the water, be more tissue left per area in the unknown sample than in the mimetic tissue model. We did, however, also show for DESI that the measured analyte intensity was independent of the section thickness, at least within a range of 10–40 µm, despite the thicker sections having a higher concentration per area than the thinner sections (all sections having the same tissue concentration in µg/g). What appeared to matter was the tissue concentration in µg/g rather than the surface concentration in µg/mm^2^. In this study, MALDI is used rather than DESI with a section thickness of 20 µm, but a previous study does not suggest that thicker sections (and thus more analytes below the ionization spot) should lead to a greater signal [22]. Overall, we consider the benefits from constructing the calibration model from skin (such as a better mimicking of the sample matrix) are worth the compromise of having to add 150% water, and believe that this model is a more accurate imitation of the conditions in the actual sample as opposed to droplet deposition on skin or the use of a surrogate mimetic tissue model.

For the bleomycin in porcine skin, a limit of detection around 5–15 µg/g was estimated based on the mimetic tissue model and measured on the QExactive mass spectrometer with a SMALDI5 ion source. In comparison, in a very recent study cyclosporine was measured by DESI-MSI also on a QExactive mass spectrometer, and a mimetic tissue model of cyclosporine in a rat liver homogenate showed a limit of detection around 5 µg/g [23]. Both bleomycin and cyclosporine are peptides with molecular weights of 1415 g/mol and 1202 g/mol, respectively, but for a peptide cyclosporine has a quite low polarity compared to bleomycin.

### 3.2. Introduction of the Internal Standard

Figure 3 shows an example where the sample geometry is not perfectly aligned, causing a change in laser focus over the width of the image. The drift is clearly observed in the calibration curve (the points are by turns above or below the curve, corresponding to the left and the right side of the image), and the normalization relative to an endogenous lipid provides an excellent correction, improving the regression coefficient from 0.696 to 0.997. In the calibration curve in Figure 5, the linearity is already quite good, with the exception of one point (500 µg/g), which clearly falls below the calibration curve. The regression coefficient is 0.951 and it improves to 0.977 when the internal standard signal of reserpine is used for normalization, despite the 500 µg/g signal still falling below the curve. The internal standard thus improves the performance of the model, but it is not capable of compensating for a point on the curve, which, likely due to an imperfection in the homogenate preparation, falls a little outside the curve. However, thanks to the high number of points in the standard curve (eight concentration levels), this point can be identified and checked so that it does not alter the slope of the curve in an undesirable way. This underscores the benefits of having many concentration levels in a standard curve, and of having them equidistantly distributed in the high-concentration end of the curve as in this example.

Ideally, a stable isotope labelled version of the analyte should have been used as an internal standard. When such one is not readily available (as in this study of bleomycin), it is satisfying from the improvement of the regression coefficient to observe that the internal standard is still capable of compensating for variations throughout the entire imaging experiment. More importantly, the internal standard has two other functions. Firstly, it provides a valuable run-to-run correction when the mimetic tissue model and the unknown sample are run in separate images, as in this case when the standard curve for time reasons was run at a lower spatial resolution than the unknown sample. Secondly, the internal standard corrected for tissue heterogeneities which were much more pronounced in the skin with its different layers than, for example, in the liver, which has been the object of many previous Q-MSI studies.

We found that in an MALDI-MSI experiment where a matrix sprayer is already involved in the matrix deposition, the additional step of depositing an internal standard using the same sprayer represents a relatively minor effort compared to the potential gain in the performance of the model.

### 3.3. The Performance of the Calibration Model

The calibration model enabled the assessment of tissue concentrations in much finer detail than what can be obtained by LC-MS where typically millimeter-sized pieces of tissue are being homogenized and quantified. For skin penetration studies, horizontal cryo-sections can be collected at different depths for extraction and LC-MS in order to obtain a concentration profile through the skin, as we have previously done in another bleomycin study where MALDI-MSI and LC-MS were combined to provide a more complete characterization of the distribution of bleomycin [24].

With regard to accuracy and precision, LC-MS will always perform better than MSI due to the use of chromatography, which significantly reduces the negative impact of ion suppression on the quantitative results. The sensitivity is also higher in an LC-MS study, since much more extensive sample preparation involving pre-concentration and clean-up is possible, while the sensitivity in an MSI experiment is never better than what can be measured in each individual pixel. This means that the only sample material available for analysis is what is situated in the area of some µm^2^ of tissue, while the amount of the sample that goes into an LC-MS measurement is typically from significantly larger amounts of a sample.

The limitation of LC-MS in skin studies, however, is that the skin is a very heterogenous type of tissue where the presence of features such as the sebaceous glands and hair follicles can cause dramatic changes in skin permeation from sample to sample. In an LC-MS study, this sample-to-sample variation can be observed but not readily explained, as the only results to report from the study are numbers (tissue concentrations) [16]. In comparison to this, a Q-MSI experiment will be less accurate, precise and sensitive, but the quantitative results are obtained together with images of distributions, which add an additional layer of understanding to the quantitative results and help identify possible failed experiments, which could have confounded an LC-MS study.

Skin is a tissue of major interest to the pharmaceutical industry with many pharmaceutical products relying on topical drug delivery. Given the type of information offered by MSI, is it thus obvious that topical drug delivery has been a major field of MSI applications with MALDI-MSI [12,25,26,27] as well as with DESI-MSI [28,29], and indeed skin is also among the type of samples that have been in the forefront with regard to the development of quantitative MSI methods. Bonnel et al. performed quantitative MALDI-MSI on four different drugs in human skin by means of the tissue extinction coefficient [16], as did Handler et al. for tofacitinib in porcine skin [14], while Russo et al. [15] and Handler et al. [13] used the microspotting of droplets of the standard as well as an internal standard for the quantification of terbinafine and tofacitinib in a living skin equivalent model and in porcine skin, respectively. A very recent study by Legouffe et al. stands out as it focused on the quantification of an endogenous compound, hyaluronic acid, in human skin, which being a polymer of disaccharides (with a molecular weight up to several million daltons) has to be enzymatically digested prior to MALDI-MSI detection [30]. Only the relative quantification was made of hyaluronic acid, but a calibration curve was made of hyaluronic acid by a droplet deposition on the control skin in order to test the linearity of the detection method, including the enzymatic digestion.

In addition to this, Barry et al. used a mimetic tissue model for the quantification of a small molecule drug in porcine skin [8]; however, it was not a true mimetic tissue model based on skin homogenates but on a set of calibrants of rat liver homogenates termed a *surrogate model*, originally made for the quantification of the same compound in rat liver.

A direct comparison between these approaches and the present approach based on a mimetic tissue model is not possible based on the existing studies, since the quantified compounds and the applied instrumentations are very different. Compared to other approaches, the mimetic tissue model comes closer to the state-of-the-art approach in quantitative bioanalysis by LC-MS, e.g., for the quantification of drugs in plasma, where the standard curve is produced in blank plasma, which is spiked to the relevant concentrations and undergoes the exact same sample preparation and analysis procedures as the unknown samples. The mimetic tissue model provides a better approximation to all the interactions between the analyte and the sample matrix than methods where the standards are deposited on top of the matrix rather than inside the matrix. In recent years, a mimetic tissue model has been more frequently used, also in DESI-MSI studies where it has been used to establish the limits of detection [31,32], and this development is likely to gain speed as more laboratories become acquainted with the methodology. It is particularly pronounced for mimetic tissue models that they become more efficient in use after some initial experience with their preparation. In this context, it is important that the mimetic tissue model is made of the same tissue type as the sample being analyzed rather than that of a surrogate model. Indeed, liver homogenates are much more convenient to work with than skin homogenates, but the extra work invested in a mimetic tissue model with skin is rewarded with a better quantitative performance.

As shown in this study, concentration can be assessed in several different regions of an image. In the present study, the concentration in the different selected regions of interest range between 15 and 572 µg/g, while the LC-MS quantification of an entire biopsy from the same animal study yielded an overall bleomycin concentration of 63.5 µg/g. It would be an overstatement to claim that the Q-MSI thus had been validated by LC-MS, but it is reassuring to see that the results obtained by Q-MSI and LC-MS are in the same order of magnitude. As Barry et al. point out, quantification by MSI relies on the subjective drawing of the ROI [8], and anyone who has tried to draw ROIs in an MSI software knows that the average intensities can change dramatically when small changes are made to the ROIs. As such, the average bulk tissue concentration of an LC-MS experiment is not directly comparable to the very local concentration in a tissue section of an MSI experiment, but it is fair to expect that the two types of quantifications should yield results of the same order of magnitude. The precision of approx. 25% RSD in the present study is lower than what is typically obtained in an LC-MS study, but is not critical compared to many other factors influencing a drug delivery experiment in skin such as biological variation, the presence of follicles and sebaceous glands, etc.

## 4. Materials and Methods

### 4.1. Animals

A total of four female gilt pigs provided by Bispebjerg Hospital were used in the study from which the unknown sample was sourced. The skin biopsies were taken directly after or an hour after the animals were treated with bleomycin (1500 IU of bleomycin per 100 µL), administered by an electropneumatic injection (EPI), as previously described [21,33]. The bleomycin (batch 7K062C; Baxter; Deerfield; IL; USA) was diluted in sodium chloride 0.9% (SAL) to a concentration of 15,000 IU/m. The samples were stored at −80 °C.

### 4.2. Chemicals

Bleomycin, carboxymethylcellulose sodium and 2,5-dihydroxybenzoic acid were purchased from Merck KGaA (Darmstadt, Germany), and LC-MS-grade methanol and water were purchased from VVW (Søborg, Denmark).

### 4.3. Mimetic Tissue Model Preparation

The mimetic tissue model calibration curve was performed as described by Barry et al. [11,14], with modifications. The control skin was obtained from similar female gilt pigs, which had not been exposed to bleomycin. Initially, the muscle and most of the subcutaneous fat was removed from the skin, and the hair was shaved off. From this skin, 8 mm biopsies were taken and cut into four pieces each, and approximately 0.45–0.50 g of skin was placed in homogenate tubes. Water (150% *v*/*w*) and two steel homogenization beads (4 mm) were added to the tube. The tissue was homogenized using a BeadBlaster 24 (Benchmark Scientific, Sayreville, NJ, USA). The homogenizer parameters were set to 10 cycles of 60 s, with a speed of 7.00 m/s (4260 rpm), and a 30-s interval between each cycle. A bleomycin solution was added and the homogenization was performed for another two cycles. The homogenate was moved to a dosing pipette (0.5–2.5 mL, Qosina, Ronkonkoma, NY, USA), which was subsequently placed in a hand centrifuge and spun to avoid air bubbles and to ensure an even layer of the homogenate. The dosing pipette was frozen and a thin layer of water was added on top of the homogenate, followed by freezing and a second layer of the spiked homogenate.

A homogenate was prepared for each bleomycin concentration as well as a blank mimetic tissue. The bleomycin solutions were prepared in water to obtain the final tissue concentrations of 25, 50, 100, 200, 300, 400, 500 and 600 μg/g. The tissue cylinders, as prepared above, were combined and embedded with a 5% carboxymethylcellulose gel in a mold and frozen to become a block containing all of the calibrants. The block was cryo-sectioned in 20 μm on a Leica CM3050S cryostat (Leica Microsystems, Wetzlar, Germany), thaw-mounted onto glass slides, and stored at −80 °C until the time of analysis.

### 4.4. Cryo-Sectioning of Skin Biopsy

An 8 mm pig skin biopsy treated with bleomycin administered by an EPI was embedded in a hydroxypropyl-methylcellulose (HPMC, 7.5%) and polyvinylpyrrolidone (PVP, 2.5%) gel and stored at −80 °C. The cross-sections were made with a thickness of 20 μm. The chamber temperature was set to −20 °C, while the sample was held at −18 °C. The samples were thaw-mounted onto microscope slides and stored at −80 °C until the time of analysis.

### 4.5. MALDI-MSI

The samples were dried in a vacuum desiccator for 15 min before the analysis. A 30 mg/mL solution of 2,5-dihydroxybenzoic acid dissolved in methanol/water 70:30 was sprayed on the samples using an iMatrixSpray sprayer [34]. The spray parameters were set to 12 cycles, with a density of 3 μL/cm^2^ each, a 90 mm/s speed, a 1 mm line pitch and an 80 mm spray height, resulting in a final surface concentration of 10.8 µg/mm^2^.

For the quantitative analysis, reserpine was used as an internal standard, which was sprayed in a concentration of 2.5 µg/mL (dissolved in methanol/water 90:10) to obtain a surface concentration of 1.6 ng/mm^2^. 

An MALDI-MSI analysis was performed on a Thermo QExactive Orbitrap mass spectrometer (Thermo Fisher Scientific GmbH, Bremen, Germany) equipped with an AP-SMALDI5 ion source (TransMIT GmbH, Giessen, Germany). The analysis was carried out in positive ionization mode at a mass resolution of 140,000@*m*/*z* 200. The scan range was set to *m*/*z* 400–1600, and a DHB matrix peak was used as a lock mass to ensure a mass accuracy of ≤2 ppm. The samples were imaged with pixel sizes of 50, 150 or 200 µm in line scan mode. The bleomycin was present in the mass spectra in the forms of bleomycin A2 and bleomycin B2 with the theoretical *m/z* values 1414.51826 and 1425.56323, respectively, and the study was based on the signal of bleomycin A2. The endogenous lipid phosphatidyl choline (34:1) was detected as its sodium adduct at *m/z* 782.5672. An example of a mass spectrum from a section of skin containing bleomycin is shown in Figure 6e.

### 4.6. Data Conversion and Analysis

The raw data were converted to imzML using RAW + UDP to IMZML software (v1.6R170; TransMIT GmbH, Giessen, Germany) (19). All MSI data analyses were performed in MSiReader 1.01 [35].

## 5. Conclusions

The present study is part of a trend where MSI is moving from being primarily a qualitative technique, which must be accompanied by LC-MS when quantification is desired, to becoming a technique which provides localization and quantification in the same experiment. Compared to bioanalysis by LC-MS, Q-MSI is still in its infancy—many Q-MSI methods have been presented, but no conventions exist yet for how Q-MSI must be performed. In this process, however, the mimetic tissue model stands out like a model delivering very high similarities between the calibrants and the samples and with fewer assumptions needed compared to the various methods based on droplet deposition.

A tissue mimetic model was presented in 2013, and not until now has its use on skin samples been devised, despite skin being among the types of samples most frequently studied by MSI. The model was tested on a skin biopsy from an in vivo animal experiment with a novel injection technique and the results obtained by Q-MSI were of the same order of magnitude as what was obtained by LC-MS from a similar biopsy in a previously published study [21]. The mimetic tissue model, which after some practice takes a few hours to prepare, is more time consuming than a one-time experiment with droplet deposition. It may thus be deemed too laborious for the assessment of the limits of detection, but for an MSI study involving the quantitative imaging of several samples, it would be the most efficient and accurate way to perform a quantitative MSI of skin. In order to account for the different layers of skin, an internal standard was introduced, and it was seen from the linearity of the calibration curve that the internal standard also corrected for the signal drift. In the imaging of small molecule drugs, a stable isotope labelled version of the drug might be available, and this would provide a further improvement of the overall analysis method. The calibration model was developed with MALDI-MSI, but there is good reason to believe it should work equally well with DESI-MSI.

This study showed a run-to-run precision around 25%, a number which is not only method dependent but to a great extent also depends on the instrumentation being used. The precision may be lower than what can be obtained in some LC-MS experiments, but it is still of a scale that makes it relevant to drug delivery studies and provides a dramatic increase in the versatility of MSI.

## Data Availability

Representative data files from analysis of skin sample and mimetic tissue models are available on the Metaspace annotation platform (metaspace2020.eu). Additional data is available from the corresponding author upon request.
